# Loss of a single N-linked glycan from the hemagglutinin of influenza virus is associated with resistance to collectins and increased virulence in mice

**DOI:** 10.1186/1465-9921-10-117

**Published:** 2009-11-23

**Authors:** Patrick C Reading, Danielle L Pickett, Michelle D Tate, Paul G Whitney, Emma R Job, Andrew G Brooks

**Affiliations:** 1 Department of Microbiology and Immunology, The University of Melbourne, Parkville, 3010, Victoria, Australia; 2 WHO Collaborating Centre for Reference and Research on Influenza, North Melbourne, 3051, Victoria, Australia

## Abstract

**Background:**

Glycosylation on the globular head of the hemagglutinin (HA) protein of influenza virus acts as an important target for recognition and destruction of virus by innate immune proteins of the collectin family. This, in turn, modulates the virulence of different viruses for mice. The role of particular oligosaccharide attachments on the HA in determining sensitivity to collectins has yet to be fully elucidated.

**Methods:**

When comparing the virulence of H3N2 subtype viruses for mice we found that viruses isolated after 1980 were highly glycosylated and induced mild disease in mice. During these studies, we were surprised to find a small plaque variant of strain A/Beijing/353/89 (Beij/89) emerged following infection of mice and grew to high titres in mouse lung. In the current study we have characterized the properties of this small plaque mutant both *in vitro *and *in vivo*.

**Results:**

Small plaque mutants were recovered following plaquing of lung homogenates from mice infected with influenza virus seed Beij/89. Compared to wild-type virus, small plaque mutants showed increased virulence in mice yet did not differ in their ability to infect or replicate in airway epithelial cells *in vitro*. Instead, small plaque variants were markedly resistant to neutralization by murine collectins, a property that correlated with the acquisition of an amino acid substitution at residue 246 on the viral HA. We present evidence that this substitution was associated with the loss of an oligosaccharide glycan from the globular head of HA.

**Conclusion:**

A point mutation in the gene encoding the HA of Beij/89 was shown to ablate a glycan attachment site. This was associated with resistance to collectins and increased virulence in mice.

## Background

Mammalian serum and respiratory fluids contain a complex mixture of proteins, some of which can inhibit hemagglutination activity or neutralize the infectivity of influenza viruses. Three classes of such inhibitors have been reported. The α and γ inhibitors are sialylated glycoproteins that act as receptor analogues, binding to the receptor-binding site of influenza virus hemagglutinin (HA) to block access to cellular receptors. The β inhibitors are not receptor analogues, do not contain sialic acid and act via a mechanism distinct to that of α and γ inhibitors.

Studies by Anders *et al*. demonstrated that the β inhibitors in bovine and mouse serum were mannose-binding lectins of the collectin family [1]. Collectins are large multimeric proteins that bind to glycoconjugates rich in D-mannose and N-acetylglucosamine in a Ca^2+^-dependent manner and play an important role in innate host defence against a range of microbial pathogens (reviewed by [2,3]). Members of the collectin family include the serum mannose-binding lectin (MBL), bovine serum proteins conglutinin and collectin-43 (CL-43) and lung surfactant proteins A (SP-A) and D (SP-D). For influenza viruses of the H3 subtype, the oligosaccharide side-chain at the tip of the HA spike was shown to be critical in determining the sensitivity of the virus to the antiviral activities of collectins in mouse and bovine serum [1]. Mutant viruses selected in the presence of bovine serum (a rich source of conglutinin) were shown to have lost this glycosylation site and were resistant to hemagglutination inhibition by β inhibitors [1].

Since their identification as β inhibitors, the role of collectins in innate host defence against influenza viruses has become an area of intense interest. MBL, conglutinin, CL-43 and SP-D all act as classic β inhibitors, binding in a Ca^2+^-dependent manner to oligosaccharides expressed on the viral HA and NA glycoproteins. This mediates hemagglutination inhibition, neutralization, virus aggregation and opsonization of virus to promote neutrophil responsiveness to the virus (reviewed by [2,4,5]). In contrast, the collectin SP-A is a sialylated glycoprotein and therefore acts as a γ inhibitor to mediate a similar range of antiviral activities against influenza viruses [6,7]. Of particular interest, both SP-A and SP-D are present in respiratory secretions, although current evidence suggests that the high avidity interaction between SP-D and carbohydrates on the viral HA is a major factor contributing to the neutralizing capacity of bronchoalvolar lavage fluids [8-10].

Since their appearance in the human population in 1968, H3N2 subtype viruses have shown a progressive increase in N-linked glycosylation in and around the globular head of the HA molecule, while glycosylation sites located in the stem region of HA tend to be highly conserved [11,12]. Using a mouse model of influenza infection, we have demonstrated that for viruses of the H3 subtype (1968-1992), the level of glycosylation on the globular head of HA of a particular virus strain inversely correlates with its ability to replicate *in vivo *[8]. Virus strains bearing high levels of glycosylation (1977-1992) were more sensitive to neutralization by murine collectins, and this in turn correlated with a poor ability to replicate in mouse lung. In initial studies we were surprised to find that one virus strain, A/Beijing/353/89 (Beij/89), did not fit this trend and grew well in mouse lung despite the presence of 4 potential sites of N-linked glycosylation on the globular head of HA. Studies were therefore undertaken to determine the mechanisms underlying the enhanced virulence of this particular mutant for mice.

## Methods

### Viruses

A seed stock of wild-type (non-reassortant) A/Beijing/353/89 (Beij/89) from the WHO Collaborating Centre for Reference and Research on Influenza, Melbourne, Australia and was propagated once at a 10-4 dilution in the allantoic cavity of 10-day embryonated eggs to generate an uncloned stock of Beij/89. When this stock was plaqued on MDCK cell monolayers in the presence of trypsin [8], two morphologically distinct plaque types were observed; a predominant round plaque type approximately 1 mm in diameter (large plaque phenotype), and a minor subpopulation (<5%) of small, star-shaped plaques (small plaque phenotype).

Plaque purification (PP) of virus was performed on MDCK cells and was monitored by the distinctive plaque morphology of the large and small plaque viruses. Well-separated plaques were picked, resuspended in PBS and inoculated into 10-day embryonated hens' eggs. Allantoic fluid was harvested and the PP procedure repeated. Stocks of allantoic fluid generated from the second PP were plaqued to ensure appropriate morphology and frozen at -70°C. Purified virus stocks were prepared using discontinuous sucrose gradients as described [1].

### Infection and treatment of mice

C57BL/6 mice were bred and maintained in the animal facility of this department. Adult mice (6-8 weeks) were used in all experiments. All research complied with the University of Melbourne's Animal Experimentation Ethics guidelines and policies. Mice were anaesthetized and infected intranasally (i.n.) with 10^5 ^PFU of influenza virus (unless otherwise stated) in 50 μl of PBS. Each day, mice were weighed individually and monitored for signs of illness. To determine viral titres, mice were euthanized and lungs and nasal tissues were removed and homogenates were clarified by centrifugation. The samples were assayed for infectious virus by plaque assay on MDCK monolayers [8].

### Differential leukocyte counts in bronchoalveolar lavage (BAL) fluids

For collection of BAL cells, mice were killed and the lungs flushed three times with 1 ml of PBS through a blunted 23-guage needle inserted into the trachea. Cells were treated with Tris-NH_4_Cl (0.14 M NH_4_Cl in 17 mM Tris, adjusted to pH 7.2) to lyse erythrocytes, washed in RPMI 1640 medium supplemented with 10% FCS and cell viability was determined via trypan blue exclusion. For differential counts, aliquots of approximately 5 × 10^4 ^BAL cells were cyto-centrifuged onto glass microscope slides, dried and stained with Diff Quick (Lab Aids, Australia). Slides were examined using a light microscope and a minimum of 100 cells in 4-8 random fields was counted (×1000 magnification). Macrophages, lymphocytes and neutrophils were identified by their distinct nuclear morphologies.

### Sera, mAbs and SP-D

Mouse serum was collected from blood that had clotted at 4°C overnight followed by storage at -70°C. Recombinant rat SP-D was a gift from Prof. Erika C. Crouch, Department of Pathology, Washington University School of Medicine, St. Louis, Missouri, USA. The anti-HA mAbs CY3/3, PA1/1 and C1/1 raised against BJx109 (A/Beijing/353/89 × A/PR/8/34) were prepared by Dr. Georgia Kapakalis-Deliyannis, Department of Microbiology and Immunology, University of Melbourne. mAb D7/1, raised against A/Philippines/2/82 (Phil/82), was prepared by Dr. E. M. Anders, Department of Microbiology and Immunology, University of Melbourne.

### Virus Neutralization assays

Neutralization of virus infectivity was measured by fluorescent-focus reduction in monolayers of MDCK cells cultured in 96-well plates (Nunc, Golstrup, Denmark) as described [8]. Briefly, dilutions of mouse sera or recombinant rat SP-D were mixed with a constant dilution of virus, and after incubation for 30 mins at 37°C, added to MDCK cell monolayers. After adsorption of virus for 45 min at 37°C, the inoculum was removed and cells were incubated a further 7-8 hrs to allow for infection of MDCK cells. Cell monolayers were then fixed in 80% acetone and stained for fluorescent foci by incubation with mAb A-3, specific for the nucleoprotein (NP) of type A influenza viruses, followed by fluorescein-conjugated rabbit anti-mouse immunoglobulins (Silenus, Melbourne, Australia).

### Hemagglutination and Hemagglutination Inhibition (HI) assays

Hemagglutination titrations and HI tests were performed by standard procedures using 1% (vol/vol) chicken erythrocytes in Tris-buffered saline (TBS; 0.05 M Tris-HCl, 0.15 M NaCl, pH 7.2) containing 0.1% NaN_3 _(TBSN_3_).

### Sequencing of HA gene

Influenza virus RNA was extracted directly from allantoic fluid. Virus was digested with proteinase K and 0.5% sodium dodecylsulfate (SDS) and heated to 55°C for 5 min. RNA was extracted using hot phenol, followed by phenol-chloroform extraction and ethanol precipitation. Full length HA cDNA was prepared from viral RNA using AMV reverse transcriptase (Promega, U.S.A.). Two segments were then amplified from the HA gene PCR for direct sequencing. Sequences were determined using a PRISM Ready Reaction Dyedeoxy terminator cycle sequencing kit (Perkin Elmer, Applied Biosystems Division, Foster City, CA, USA). The complete sequence of HA for the L phenotype virus has been deposited in GeneBank (U97740).

### SDS-PAGE and immunoblot for HA

Proteins from purified preparations of influenza virus were resolved by SDS-PAGE (5-12.5% gradient gels) under non-reducing conditions, transferred to nitrocellulose and probed with 1/500 dilution of ascitic fluid of mAbCY3/3 in TBS containing 2.5 mg/ml BSA. After washing, bound antibody was detected with 1/400 dilution of HRP-conjugated rabbit anti-mouse immunoglobulins (Dako, Glostrup, Denmark). SeeBlue pre-stained standards (Novex, San Diego, California) were used to estimate molecular weights.

## Results

### Small plaque mutants of Beij/89 emerge following intranasal infection of mice

In previous studies, we have used viruses of the H3 subtype (1968-1992) to examine the relationship between the degree of glycosylation of the HA glycoprotein, the sensitivity to the antiviral activities of collectins and the ability of a particular virus strain to grow in mouse lung [8]. Strains isolated post-1980 were found to bear high levels of glycosylation on the HA, were highly sensitive to neutralization by SP-D and MBL *in vitro *and replicated poorly in the lungs of mice following intranasal inoculation. In these studies we were surprised to find that inoculation of mice with one virus strain, Beij/89, lead to sporadic growth of virus in mouse lung and that the plaques derived from the lung homogenates were noticeably different to those observed when plaquing the virus inoculum.

On closer inspection, we observed a small proportion (less than 5% of >300 plaques counted) of small plaque variants present in the original seed stock; therefore we picked 5 large (clones L1-5) and five small (clones S1-5) plaques, repeated the plaque purification and propagated each clone individually in hens' eggs. Mice were inoculated with the seed stock of Beij/89 and 3 days later the phenotype of virus present in lung homogenates was examined following plaque assay on MDCK cells. Five plaques (all of small plaque phenotype) were picked and propagated individually in hens' eggs (clones Mo1-5). All plaques of the L phenotype were significantly larger than either S or Mo plaques (1300 +/- 100 μm for L virus compared to 326 +/- 17 μm and 325 +/- 19 μm for S and Mo viruses, respectively. n = > 10 plaques measured for each sample). Together, these findings suggest that a mouse virulent variant of Beij/89 was present at low frequency in the seed stock of Beij/89 and that this variant is rapidly selected to become dominant following intranasal infection of mice.

### Enhanced replication of small plaque mutants of Beij/89 in the respiratory tract of mice

**Figure 1:**
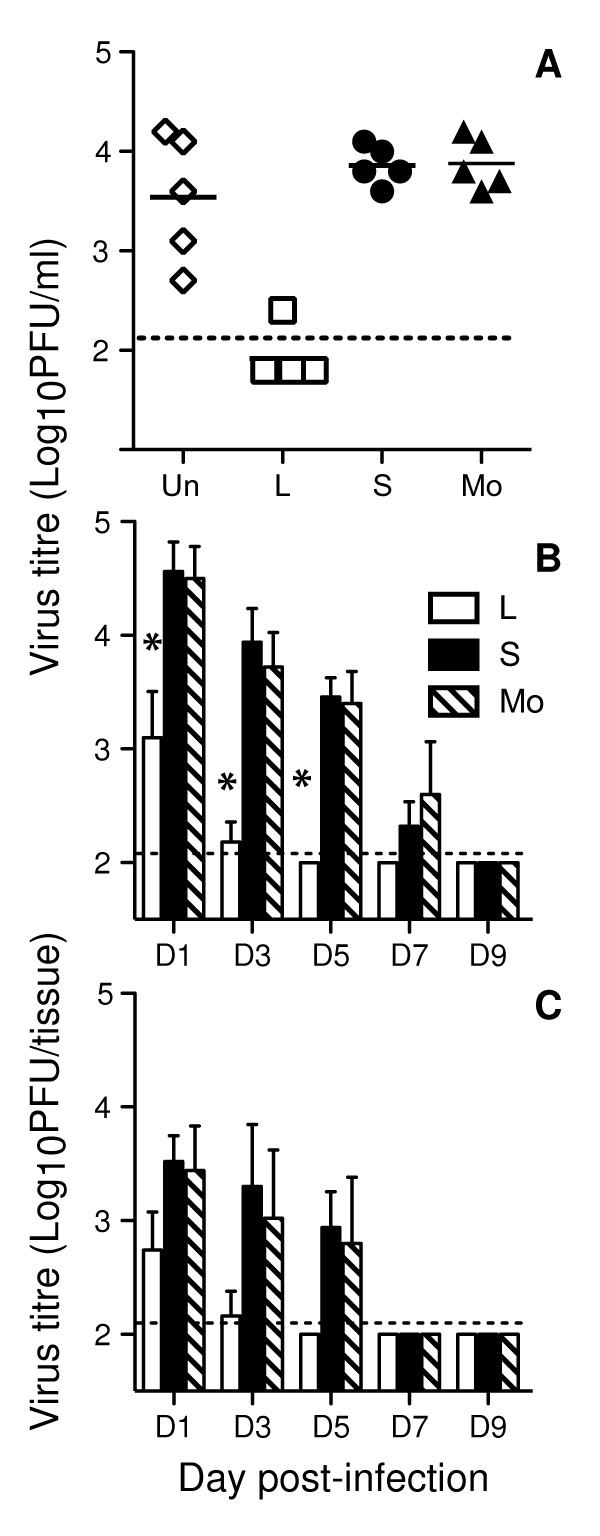
**Small plaque variants of Beij/89 show enhanced virulence for mice**. (A) Replication of uncloned Beij/89 (Un) and plaque purified L, S or Mo viruses in mouse lung. Data shown represent virus titres in the lungs of 5 mice inoculated 3 days prior with 10^5 ^PFU of virus. Data was confirmed with 5 independent clones of L, S and Mo viruses. (B & C) Time course of virus titres recovered from the respiratory tract of mice following intranasal inoculation with 10^5 ^PFU of L, S or Mo viruses. Infectious virus present in (B) lung or (C) nasal tissues was determined at various times post-infection by plaque assay on MDCK cell monolayers. Virus titres are shown as the mean ± 1 SD of 5 mice. The dashed line represents the lower limit of detection of virus in each sample (2.1) and for statistical analysis samples with a value below the limit of detection were assigned an arbitrary value of 2.0. * = *p *< 0.01, Kruskal-Wallis test.

Plaque purified clones of L, S and Mo viruses were compared for their ability to replicate in mouse lung (Fig. 1A). Mice were inoculated with 10^5 ^PFU of the uncloned stock of Beij/89 or with an equivalent dose of L, S and Mo viruses and, 3 days later, mice were killed and titres of infectious virus in lung homogenates were determined using a standard plaque assay. Variable virus titres were recorded in the lungs of mice infected with uncloned Beij/89 while virus titres were very low or undetectable in lung homogenates from mice infected with L plaque virus. Of interest, virus titres were 10-100 fold higher in the lungs of mice infected with either S or Mo viruses.

We next examined the time course of viral replication using 1 representative clone of each of the L, S and Mo Beij/89 viruses. Compared to L virus, S and Mo viruses replicated to higher titres in both the lungs (Fig. 1B) and the nasal tissues (Fig. 1C) of infected animals at all time-points examined. Virus could not be detected in the lungs of mice infected with the L phenotype virus after day 3 post-infection but was recovered from most animals infected with S or Mo viruses up to 7 days after infection (8/10 animals infected with S virus, 7/10 animals infected with Mo virus at day 7 post-infection), indicating a marked delay in clearance from the lungs. A similar trend was observed in the upper respiratory tract with delayed clearance of S and M viruses compared to L virus-infected mice (Fig. 1C).

### Airway inflammation is exacerbated in mice infected with small plaque mutants of Beij/89

**Figure 2:**
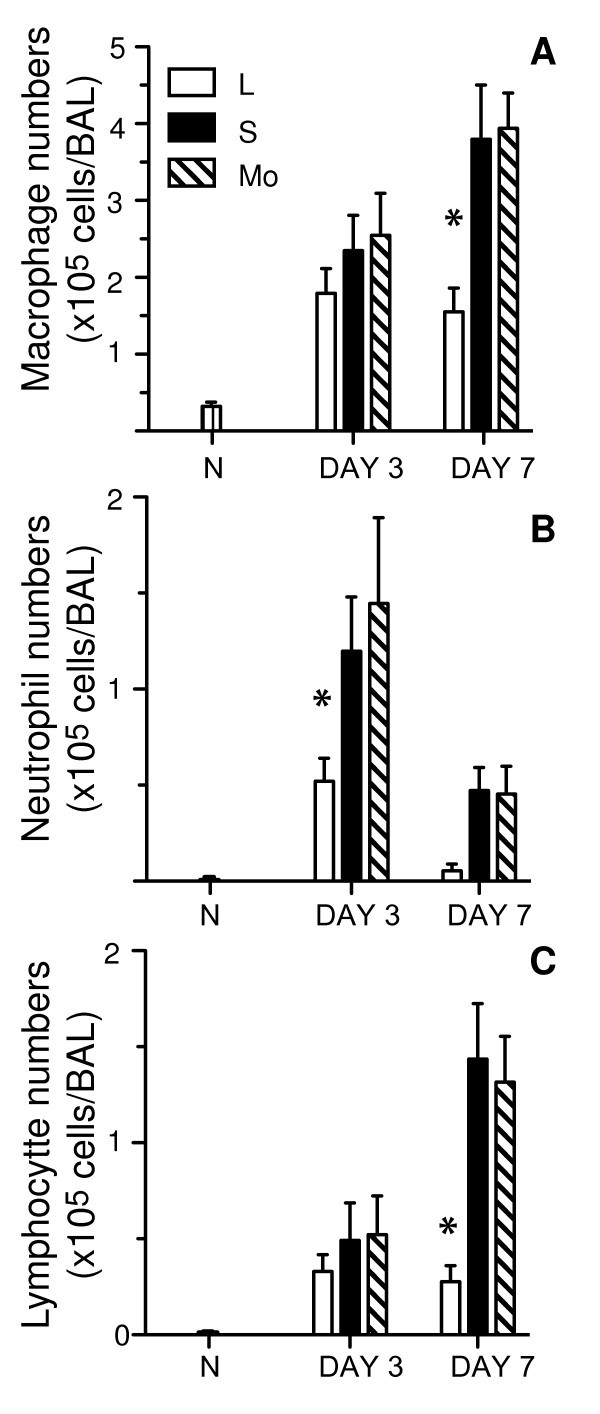
**Increased recruitment of inflammatory cells to the airways of mice infected with small plaque variants of Beij/89**. Mice were infected with 10^5 ^PFU of L, S or Mo viruses and, at day 3 or day 7 post-infection, mice were killed and BAL performed. BAL cells were centrifuged onto glass slides, stained with Diff Quick and the number of macrophages, neutrophils and lymphocytes were determined by nuclear morphology. Cell numbers from BAL of naïve (N) mice are included for comparison. Data represent the mean (± 1 SD) number of (A) macrophages, (B) neutrophils or (C) lymphocytes from groups of 5 mice and are representative of 2 independent experiments. * = *p *< 0.01, Kruskal-Wallis test.

To assess the acute inflammatory response to infection, mice infected with L, S or Mo viruses were killed at 3 and 7 days post-infection, and the cells recruited to the airspaces of the lung were recovered and characterized by differential staining. Macrophages were the predominate cell type recovered in the BAL at day 3 and 7 post-infection (Fig. 2A), and numbers were significantly higher in the lungs of mice infected with S or Mo viruses at day 7 post-infection compared to mice infected with L virus. Few neutrophils were present in the BAL of naive mice (N; < 1% of all BAL cells) but rose markedly at day 3 post-infection and declined thereafter (Fig. 2B), consistent with their role in innate host defence against influenza virus. Mice infected with S or Mo viruses had significantly higher neutrophil BAL counts compared to L virus-infected mice at both day 3 and 7 post-infection. At day 7, a significant influx of lymphocytes was observed in BAL of mice infected with S and Mo viruses (Fig. 2C), that was not observed in the airways of L virus-infected animals. Together, these data indicate that infection of mice with S or Mo viruses leads to enhanced recruitment of inflammatory cells to the lungs during the early (day 3) and latter (day 7) phases of infection.

### Small plaque mutants of Beij/89 are less sensitive to neutralization by murine collectins

**Figure 3:**
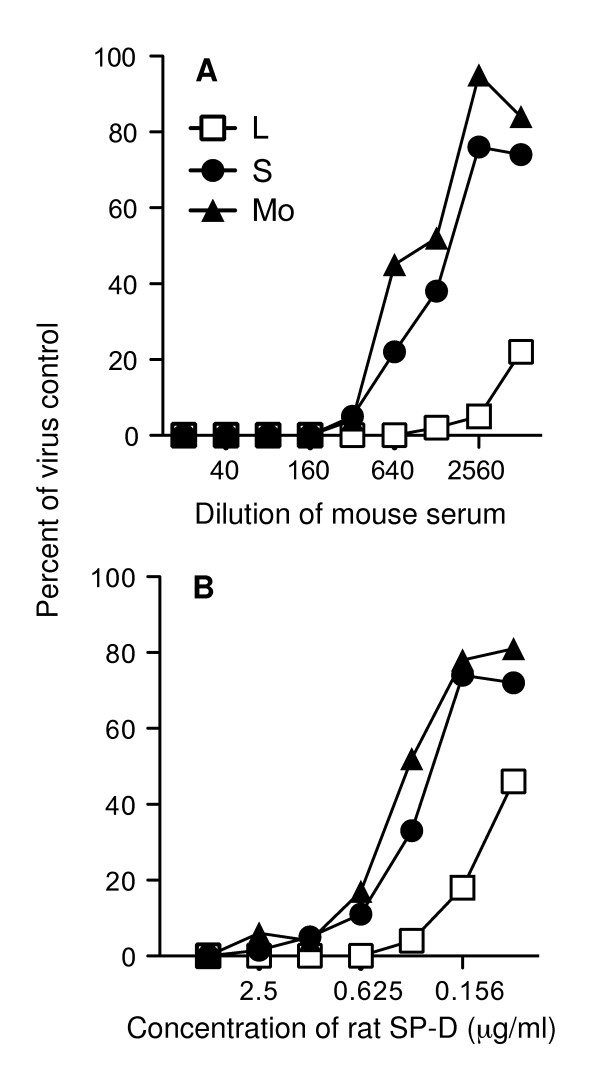
**Neutralization of plaque purified L, S and Mo clones of Beij/89 by the mannose-binding lectin in normal mouse serum and recombinant rat SP-D**. L, S and Mo viruses were incubated with dilutions of (A) mouse serum or (B) rat SP-D for 30 mins at 37°C, and the amount of infectious virus remaining was determined by fluorescent focus assay. The total number of fluorescent foci in four representative fields were counted and expressed as a percentage of the number of foci in the corresponding area of duplicate control wells infected with virus alone ('Percent of virus control'). The neutralizing activity of 1/100 dilution of mouse serum or 1 μg/ml rat SP-D was inhibited by addition of 100 mM mannose to serum or SP-D prior to addition of virus (data not shown) demonstrating that the major neutralizing activity in each sample was a mannose-specific lectin.

Collectins function as β inhibitors against influenza viruses, binding in a Ca^2+^-dependent manner to mannose-rich glycans at the tip of the HA spike resulting in steric hindrance of binding of the viral HA to host cell receptors [1,13]. Early studies demonstrated that H1 subtype viruses that had undergone adaptation resulting in increased growth in mice had also developed resistance to HI by β inhibitors [14,15], consistent with an important role for collectins in innate host defence. Therefore, we tested S, L and Mo viruses for their sensitivity to hemagglutination inhibition by rat SP-D or by MBL in mouse serum. Both SP-D and MBL were able to mediate HI activity against L, S and Mo viruses, and activity was Ca^2+^-dependent and blocked by the sugar mannose (data not shown). Viruses did, however, differ in their sensitivity to both SP-D (Fig. 3A) and MBL (Fig. 3B) in a neutralization assay. L viruses were markedly more sensitive to neutralization by either murine collectin when compared to S and Mo viruses. The neutralizing activity of SP-D or MBL against L, S or Mo viruses was abrogated in the presence of 50 mM D-mannose (data not shown).

### Loss of an N-linked glycan from the HA of small plaque variants of Beij/89

**Figure 4:**
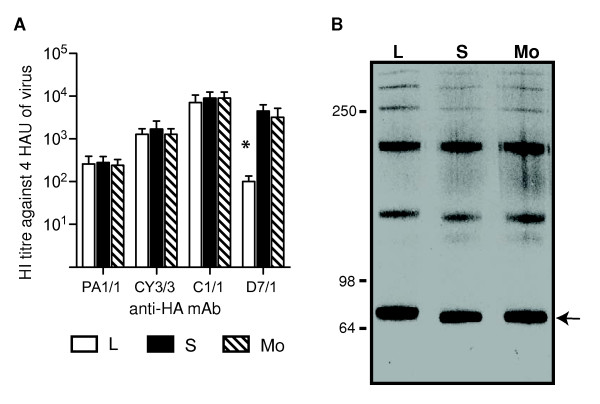
**Loss of a N-linked glycosylation site from the HA of S and Mo clones of Beij/89**. (A) HI titres of anti-HA monocloncal antibodies against L, S and Mo clones of Beij/89. HI titres are expressed as the recipricol of the highest dilution of ascites fluid to inhibit 4 hemagglutinating units (HAU) of virus. Data presented are the mean of 3 independent experiments and are representative of results obtained with 3-5 independent clones of L, S or Mo viruses. * = *p *< 0.01, Kruskal-Wallis test. (B) Immunoblot showing the HA proteins of plaque-purified L, S and Mo clones of Beij/89. Proteins from purified viruses were resolved by SDS-PAGE under non-reducing conditions on a 5-10% gel and transferred to a nictrocellulose membrane. To detect the HA glycoproteins, the blot was incubated with anti-HA mAb CY3/3 and developed, as described in Materials and Methods. The location of the HAO monomer at approximately 80 kDa is indicated with an arrow.

MBL in mouse serum and rat SP-D bind to the HA and NA glycoproteins of influenza virus, with the majority of binding to the HA molecule [8]. Given the reduced sensitivity of S and Mo viruses to neutralization by murine collectins, we compared viruses for their reactivity to a panel of HA-specific mAbs to determine if any antigenic changes could be detected in HA (Figure 4A). L, S and Mo viruses reacted equally well with mAbs PA1/1, CY3/3 and C1/1 however S and Mo viruses showed a 30-fold higher HI titre that did L viruses against mAb D7/1. The enhanced reactivity of mAb D7/1 with S and Mo viruses suggests that amino acid changes common to the small plaque mutants of Beij/89 affect the antigenic epitopes recognized by this mAb.

To determine if the reduced sensitivity of S and Mo viruses to neutralization by murine collectins was associated with loss of a glycosylation site from the HA molecule, proteins from purified virus preparations were resolved by SDS-PAGE under non-reducing conditions and the HA glycoprotein was detected by western blot using mAb CY3/3. Under these conditions, the HA runs as a series of bands corresponding to monomers, dimmers, trimers and higher molecular weight forms of the 80 kDa molecule (Fig. 4B). Loss of a glycan from HA should result in a reduction in molecular weight and therefore increased mobility of the HA on SDS PAGE. Consistent with this, the HA of both S and Mo viruses had increased electrophoretic mobility compared to that of the L virus (Fig. 4B).

To confirm loss of a glycosylation site from the HA of S and Mo viruses and to determine the location of this site, the cDNA of 3-5 isolates of L, S and Mo viruses was obtained by reverse transcription of viral genomic RNA and the complete HA_1 _and HA_2 _gene segments of each virus were amplified by PCR and sequenced. The amino acid sequence of the HA_1 _of L phenotype viruses was identical to that of A/Beijing/353/89 as determined by [16] (GeneBank L76036). The entire HA_1 _and HA_2 _sequences of S and Mo viruses were identical and differed from L viruses by a single base substitution (A^814^→T) in HA_1_, giving rise to amino acid substitution Asn to Ser at residue 246. This substitution deletes the potential glycosylation site Asn_246_-Ser_247_-Thr_248 _on the HA_1 _of the S and Mo viruses. No differences were noted in the sequence of NA or M2 proteins between clones of L, S and Mo viruses.

### L, S and Mo viruses do not differ in their ability to infect or replicate in murine respiratory epithelial cells

**Figure 5:**
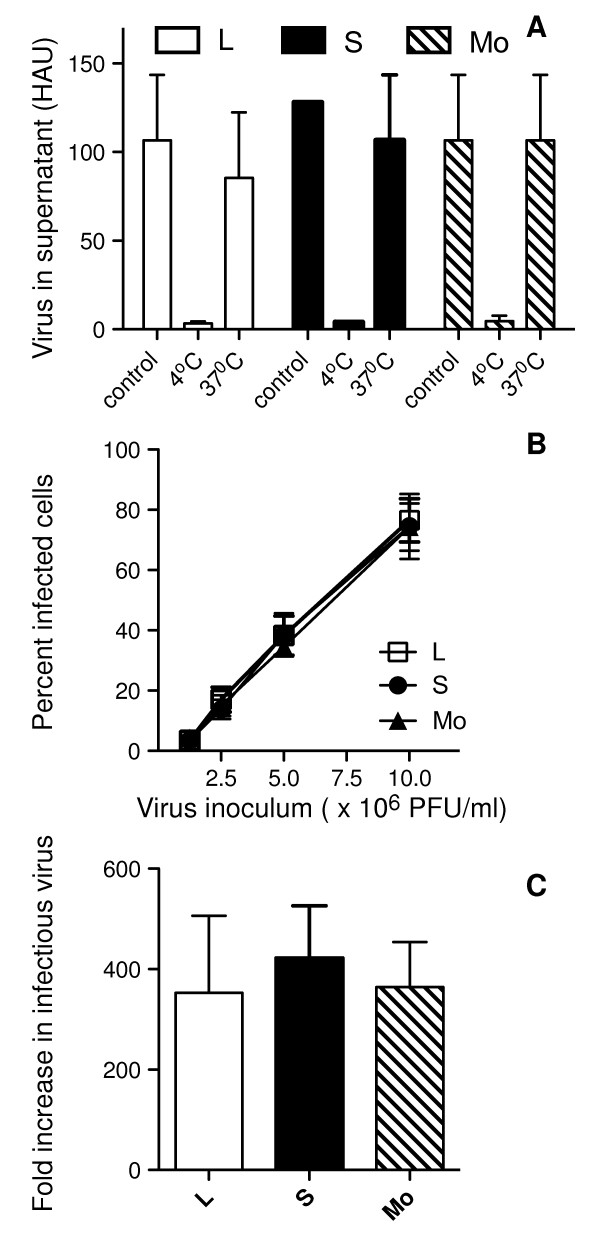
**Beij/89 viruses do not differ in their ability to bind to, infect or replicate in murine respiratory epithelial cells**. (A) Ability of viruses to bind to and elute from the epithelial cell surface. Duplicate tubes of formaldehyde-fixed LA-4 epithelial cells were mixed with 100 HAU of L (white columns), S (black columns) or Mo (striped columns) viruses for 1 hr on ice. After virus adsorption, one sample was held at 4°C ('4°C') while the duplicate tube was transferred to 37°C for 30 min ('37°C'). A third tube with no cells received virus alone ('control') and was held on ice throughout. Following centrifugation of samples, virus in each of the supernatants was quantified by hemagglutination assay. Data shown are the mean ± 1 SD HAU from 3 independent experiments. (B) Monolayers of LA-4 cells were infected in chamberslides with increasing doses of L, S and Mo viruses as described in Materials and Methods. At 6-8 hrs post-infection cells were fixed and stained via immunofluorescence for expression of influenza virus NP. Data represents the mean percent infection ± 1 SD from 4-8 independent fields per chamber. (C) Release of infectious virus from influenza virus-infected LA-4 cells. Monolayers of cells were infected with L, S or Mo viruses at a multiplicity of infection of 10 PFU/cell and, after 1 hr, washed to remove any unbound virus. Samples of supernatant were removed at 2 hrs and 24 hrs and levels of infectious virus determined by plaque assay on MDCK cells. The fold increase in infectious virus was calculated by dividing the viral titre obtained at 24 hrs post-infection by that at 2 hrs. Data are the mean ± 1 SD from 3 independent experiments.

The enhanced ability of S and Mo viruses to grow in mouse lung could relate to their ability to avoid a component of host defence, but could also reflect an inherent increased efficiency in the ability of these viruses to infect or replicate in epithelial cells lining the respiratory tract. As the balance of HA and NA activity of a virus can affect the efficiency of cell entry [17], we first compared the ability of viruses to bind and elute from LA-4 cells, a mouse respiratory epithelial cell line (Fig. 5A). In this assay, each virus was incubated, in duplicate, with fixed LA-4 cells on ice to allow virus adsorption, and then one tube was shifted to 37°C for 30 min to allow NA activity to elute virus from the cell surface. After centrifugation of samples, the virus present in the supernatant was quantified by hemagglutination assay. Each virus strain bound efficiently to LA-4 cells on ice (as indicated by reduced hemagglutinating activity), and all eluted to a similar degree when moved to 37°C (as indicated by an increase in hemagglutinating activity).

Next, we determined the susceptibility of LA-4 cells to infection by L, S and Mo viruses. In this assay, cell monolayers were incubated with increasing concentrations of L, S or M viruses and immunofluoresence performed 6-8 hrs post-infection to detect newly synthesized nucleoprotein (NP) in virus-infected cells. As seen in Fig. 5B, no significant differences were detected in the ability of viruses to infect murine respiratory epithelial cells *in vitro*, regardless of the inoculum dose used. An additional factor that could modulate viral load in the airways is the capacity of L, S and Mo viruses to replicate and release newly synthesized virions from airway epithelial cells. Therefore, monolayers of LA-4 cells were infected with 10 PFU/cell of L, S and Mo viruses, washed extensively to remove unbound virus and incubated 2 or 24 hrs before titres of infectious virus in cell-free supernatant were determined by plaque assay. As seen in Fig. 5C, viral titres increased markedly between 2 and 24 hrs, indicative of productive virus replication, however no significant differences were noted in ability of L, S and Mo viruses to replicate in these cells.

### Clearance of L virus, but not S or Mo viruses, from mice by the innate immune system

**Figure 6:**
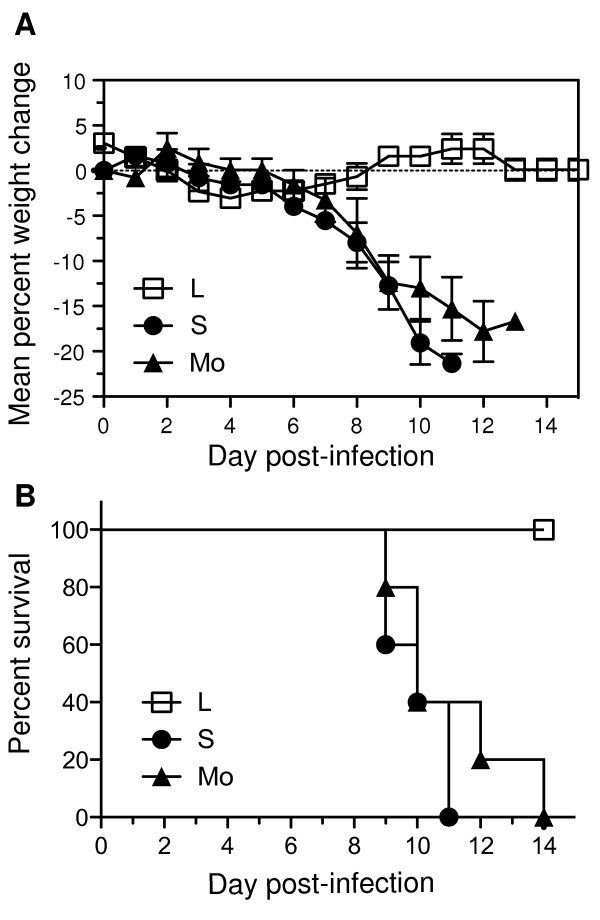
**S and Mo variants of Beij/89 are lethal for RAG1^-/- ^mice but L variants are not**. Groups of 5 mice infected with 10^5 ^PFU of Beij/89 viruses via the intranasal route were examined daily for weight loss and signs of disease. (A) Weight loss and (B) survival curves are shown for mice inoculated with L, S or Mo viruses. Mice were weighed daily and results expressed as the mean percent weight change of each group (± SEM), compared to original body weight. Animals that had lost ≥20% of their original body weight and/or presented with evidence of pneumonia were euthanized.

C-type lectins, including MBL and SP-D, play an important role in innate host defence and we have shown that S and Mo viruses are markedly more resistant to neutralization by rat SP-D and murine MBL (Fig. 3). Furthermore, B6 mice infected with L virus had cleared virus from the lung by day 5 post-infection (Fig. 1B), suggesting that infection was effectively controlled and eliminated by the innate immune system. To test this further, B6.RAG^-/- ^mice that lack T and B cells were infected with 10^5 ^PFU of L, S and M viruses and monitored daily for weight loss and signs of clinical disease. B6.RAG^-/- ^mice infected with L virus did not lose weight (Fig. 6A) did not show any signs of clinical disease (data not shown) and all mice survived infection (Fig. 6B). In contrast, mice infected with S or M viruses lost weight progressively, developed clinical disease (rapid breathing, huddling behaviour, lethargy) and all mice succumbed to infection within 8-14 days.

## Discussion

Members of the collectin family can function as β inhibitors of influenza virus and have been implicated in playing an important role in innate host defence during influenza infections. For H3 subtype influenza viruses, sensitivity to β inhibitors has been shown to correlate with the presence of a mannose-rich oligosaccharide at residue 165, located at the tip of the HA spike. In this study we have identified a second glycosylation site at residue 246 on the globular head of H3 subtype viruses that enhances sensitivity to collectins and modulates virulence for mice. Loss of glycosylation site 246 was not associated with classical resistance to β inhibitors when assessed by HI assay, however mutant viruses lacking this site were shown to be more resistant to neutralization by murine MBL and SP-D *in vitro*. Mutant viruses showed no difference in their ability to replicate in respiratory epithelial cells *in vitro*, but were markedly more virulent following intranasal inoculation of mice. These studies provide further evidence that multiple glycosylation sites on the HA of H3 subtype influenza viruses are involved in determining sensitivity to the antiviral activities of collectins both *in vitro *and *in vivo*.

In previous studies we have reported a strong correlation between the degree of glycosylation of the influenza virus HA, sensitivity of particular virus strains to neutralization by collectins and their virulence in mice [8]. Virus strains of the H3 subtype isolated after 1982 were particularly sensitive to neutralization by murine SP-D and MBL *in vitro *and were very poor in their ability to replicate in mouse lung. Compared to earlier virus strains, Phil/82, Beij/89 and Beij/92 had acquired an additional glycosylation site at residue 246 of HA_1 _and this site appeared to be associated with the enhanced sensitivity of these viruses to neutralization by collectins. Virus mutants selected for resistance to β inhibitors lack oligosaccharide attachments at residue 165 (H3 subtype) or 104 (H1 subtype)[1,13], yet recent evidence suggests additional glycans on the viral HA also contribute to SP-D-mediated antiviral activity. The use of reverse genetics to engineer additional sites of glycosylation into the globular head of the HA of A/Hong Kong/1/68 (H3N2) resulted in viruses that were more sensitive to inhibition by human SP-D and showed attenuated disease in mice [18]. Sequential addition of glycosylation to sites 63, 126 and 246 was associated with step-wise increases in sensitivity to SP-D. Glycosylation site 165 has been shown to carry high-mannose glycans in early H3 virus strains such as A/Aichi/2/68 and Memphis/102/72 [19,20], however Beij89 (L virus) carries additional sites of potential N-linked glycosylation at residues 63, 126, and 246 on the head of HA and, if glycosylated, the nature of specific glycans remains to be defined. The enhanced resistance to SP-D associated with loss of glycan 246 from Beij/89 is consistent with the expression of mannose-rich oligosaccharides at this site although the sugar specificity of SP-D does not exclude interactions with complex or hybrid-type glycans. Of interest, recent studies comparing H3N2 virus strains implicated additional glycan attachments at positions 122, 133 and 144 in contributing to the enhanced sensitivity of post-1995 H3N2 subtype viruses to human SP-D [21].

Inhibition of the hemagglutinating activity of influenza viruses represents a simple and convenient assay for determining sensitivity to β inhibitors by assessing the ability of the inhibitor to block access of viral HA to sialylated erythrocyte receptors. For H3 subtype viruses, the presence of a mannose-containing oligosaccharide at residue 165 has been shown to be critical for determining sensitivity to HI by β inhibitors in bovine and mouse serum [1], as well as by purified SP-D [22]. The current study indicates that additional glycosylation sites on HA, such as that at residue 246 on the HA of Beij/89, also play an important role in other antiviral activities mediated by collectins, such as neutralization of virus infectivity. Due to the multimeric nature of collectins, additional glycosylation sites on the head of HA presumably facilitates an enhanced degree of binding, thereby strengthening the overall affinity with which a collectin can bind to and inactivate virus. Previously, a mutant H3N2 virus selected for resistance to rabbit serum was found to have lost a potential glycosylation site at residue 246 on HA_1 _[23] and subsequent studies demonstrated that the inhibitor in rabbit sera was a mannose-binding lectin [24]. Our study has extended these findings to demonstrate the critical role of this glycosylation site in determining sensitivity to murine SP-D, which is found in airway secretions, and in modulating virulence in a mouse model of influenza infection.

The presence of the small plaque mutant in the seed stock of Beij/89 suggests that loss of the glycosylation site at residue 246 on the HA_1 _of Beij/89 confers a growth advantage upon cultivation of virus in eggs. Egg adaptation has been associated with a number of sequence changes that alter potential N-linked glycosylation sites on the HA, including the site at residue 246 of H3 subtype viruses [25-27] although loss of this site from particular H3 field strains was not associated with adaptation to growth in eggs [16]. In general terms, amino acid substitutions associated with egg-adapted variants cluster around the receptor-binding site on the HA molecule [28] and are likely to increase binding to Sia(α2,3)Gal moieties on cells of the chicken embryo chorio-allantoic membrane, the site of influenza virus replication in chicken eggs. N-linked glycans in the vicinity of the receptor-binding site have been proposed to sterically interfere with binding to Sia(α2,3)Gal-containing macromolecules and so may be lost during adaptation to growth in eggs [29].

In studies not presented here, L, S and Mo viruses showed no differences in their ability to agglutinate chicken erythrocytes that had been treated with increasing concentrations of periodate or *Vibrio cholerae *neuraminidase to oxidize or remove sialic acid residues, nor in their ability to agglutinate erythrocytes from a variety of avian or mammalian species (chicken, turkey, monkey, guinea pig, mouse, rabbit, cow and human erythrocytes), and on this basis, displayed no evidence of modified receptor specificity. Subtle differences in the fine specificity of receptor binding between the Beij/89 variants that went undetected in these assays could, however, be of particular relevance during growth of viruses in eggs or in mouse lung. It is well established that Sia(α2,3)Gal moieties are expressed throughout the murine respiratory tract [30], however our findings that L, S and Mo viruses are similar in their ability to infect and replicate in murine respiratory epithelial cells (Fig. 6) suggests that changes in S and Mo mutant viruses has not altered the intrinsic ability of these viruses to replicate in the appropriate target cells. Instead, their enhanced growth in mouse lung likely represents evasion of a particular host defence mechanism.

Glycosylation site 246 is located on the globular head of HA_1 _and alterations in this site could presumably influence other functions mediated by the viral HA. While receptor binding by the viral HA is required for binding and entry of virus into target cells, efficient fusion of viral envelope with endosomal membranes in the target cell is required to initiate infection. Previous studies implicated residue 246 on the HA of A/Philippines/2/82 (H3N2) as a critical residue influencing stability of HA at low pH [13], a factor critical in inducing the conformational change of the HA to expose the fusion peptides in the acidic environment of the endosome [31,32]. However, L, S and Mo viruses were found to be similar in their ability to mediate hemolysis of human erythrocytes over a range of pH using the fusion assay described ([33], unpublished observations). Sequencing of the NA gene of multiple clones of S or Mo viruses confirmed that there were no amino acid substitutions in the NA, consistent with our findings that its enzymatic activity was not significantly different between L, S and Mo viruses when assessed by ELISA [8] (unpublished observations).

Particular stains of influenza virus appear to be intrinsically more virulent than others, due largely to their ability to cause viral pneumonia and/or predispose the host to bacterial super-infection. Factors determining the virulence of particular virus strains are not fully understood although a number of lines of evidence suggest that in humans, as in mice, glycosylation may modulate viral virulence. Neither the virulent 1918 Spanish influenza pandemic virus (H1N1) [34] nor the mouse-adapted A/PR/8/34 (H1N1) [35] virus carry potential sites for N-linked glycosylation on the head of HA and human H5N1 isolates were resistant to HI by human SP-D [21], consistent with the notion that virulent strains carry less glycosylation to evade collectin-mediated defences. Following its appearance in the human population, the H3N2 strain of 1968 contained only 2 sites on the globular head of HA, and despite the presence of oligomannose at residue 165 [20], early H3 strains were partially resistant to SP-D [8,21]. In the process of antigenic drift, the accumulation of glycosylation on the head of HA may shield the virus from pre-existing antibody in the human population [12,36] but increase sensitivity to collectin-mediated antiviral activities. While evidence indicates the maintenance of extensive glycosylation on the head of recent H3 viruses [37,38], loss of glycosylation from the HA of particular human isolates [16,39,40] argues for the existence of a fine balance between protection afforded by antibody and increased sensitivity to SP-D.

## Conclusion

Members of the collectin family function as β inhibitors of influenza virus and play an important role in innate defence during influenza infections. For H3 subtype viruses, the mannose-rich oligosaccharide at residue 165, located at the tip of the HA molecule, is a critical factor in determining sensitivity to β inhibitors. In this study, we identify a second glycosylation site on the head of the HA of virus strain Beij/89 (H3N2) that enhances sensitivity to collectins and attenuates virulence for mice. While it is well established that accumulation of glycosylation on the head of HA may shield the virus from pre-existing antibody in the human population, our findings provide evidence that glycosylation at specific sites will increase collectin binding to the viral HA. As a virus persists in the human population, a balance between protection from antibody-mediated neutralization and sensitivity to particular innate host defences is likely to determine the most favourable glycosylation pattern of a particular virus strain.

## Competing interests

The authors declare that they have no competing interests.

## Authors' contributions

PR carried out the majority of experiments described in the study, analyzed and interpreted data and wrote the manuscript. DP, MT, PW and EJ performed some of the experiments described in this study. AB contributed to interpretation of data and the writing of the manuscript. All authors read and approved the final manuscript.
